# Serum Col3-4: A new type III and IV collagen biochemical marker of synovial tissue turnover in patients with rheumatoid arthritis

**DOI:** 10.1371/journal.pone.0282954

**Published:** 2023-04-13

**Authors:** Evelyne Gineyts, Marjorie Millet, Olivier Borel, Frédéric Coutant, Jean-Charles Rousseau, Roland Chapurlat, Hubert Marotte, Patrick Garnero

**Affiliations:** 1 INSERM Unit 1033, Edouard Herriot Hospital, Lyon, France; 2 PMOLab, Edouard Herriot Hospital, Lyon, France; 3 Immunogenomics and Inflammation Research Team, University of Lyon, Edouard Herriot Hospital, Lyon, France; 4 Immunology Department, Lyon-Sud Hospital, Pierre-Bénite, France; 5 Hospices Civils de Lyon, Lyon, France; 6 INSERM U1059, LBTO Team, University of Lyon, Saint-Etienne, France; 7 Rheumatology department, Hospital Nord, CHU Saint-Etienne, Saint-Etienne, France; University of Illinois, UNITED STATES

## Abstract

The objective of this study was to develop a serum biochemical marker of the degradation of type III and IV collagens, as an index of synovium turnover, and evaluate its performance in patients with rheumatoid arthritis (RA). An enzyme-linked immunosorbent assay for serum synovial collagen fragments (Col3-4) was developed using an antibody recognizing a specific sequence from human type III collagen, which shares 70% homology with type IV collagen. Immunohistochemistry was performed to localize Col3-4 and the matrix metalloprotease MMP-9 which is upregulated in RA synovial fibroblasts in the synovial tissue from a RA patient. Serum Col3-4 was measured in patients with RA (n = 66, 73% women, mean age 62 years, median disease activity score 28 with erythrocyte sedimentation rate (DAS28-ESR) 2.6) and in sex and age matched healthy controls (n = 70, 76% women, mean age 59 years). Col3-4 immunoassay demonstrated adequate analytical performances and recognized a circulating neoepitope resulting from the cleavage of type III and IV collagens. In RA synovium tissue, Col3-4 fragments were localized in the lining layer where destructive fibroblasts are present and around blood vessels rich in type IV collagen. MMP-9 colocalized with Col3-4 staining and efficiently released Col3-4 fragments from type III and type IV collagen digestion. Serum Col3-4 was markedly increased in patients with RA (+240% vs controls, *p* < 0.0001) and correlated with DAS28-ESR (*r* = 0.53, *p* < 0.0001). Patients with RA and active disease (DAS28-ESR > 3.2, n = 20) had 896% (*p* < 0.0001) higher levels than subjects with low activity (n = 46). Serum Col3-4 is a specific and sensitive biochemical marker reflecting MMP- mediated type III and IV collagen degradation from synovial tissue. Serum Col3-4 levels are markedly increased in patients with RA, particularly in those with active disease, suggesting that it may be useful for the clinical investigation of RA.

## Introduction

Rheumatoid arthritis (RA) is an inflammatory joint disease which, in addition to severe pain and impaired mobility, ultimately leads to joint damage, including cartilage degradation and bone erosion [1]. The driving mechanism of this process is an activation of the synovial membrane, characterized by increased remodelling of its matrix, neovascularisation and over secretion of inflammatory cytokines and catabolic enzymes, including matrix metalloproteases (MMPs) [1]. Circulating biological tests which are currently used to monitor inflammation and synovial tissue activity in RA include serum C-reactive protein (CRP) which is mainly produced by the liver, and in a more investigative manner cytokines, chemokines, acute serum amyloid A, S100 proteins, the adhesion molecules ICAM-1, VCAM-1 and E-selectin, and the mediators of joint damage, MMPs, osteoprotegerin, and the receptor activator of nuclear factor-κB ligand [2]. Although they are useful to assess the inflammatory status of RA subjects, their main limitation is a lack of specificity for the synovial tissue when assessed in the serum. Consequently, their association with joint damage progression is modest and non-consistent between studies [3].

Serum and urine biochemical markers of bone, cartilage and synovial tissue matrix turnover are more specific non-invasive tests of joint tissue metabolism [2]. Available clinical data in RA showed that the association of currently available serum or urine biochemical assays with radiographic joint damage and progression are inconclusive for bone markers [4, 5]- probably because systemic levels mainly reflect whole body skeleton turnover- but are more robust with cartilage markers including those measuring the degradation products of type II collagen, the main protein of its matrix.

Because synovium tissue activation is the earliest and key driver of joint damage in RA, the development of biochemical markers reflecting its remodelling may be of great value to predict joint damage progression. However, there is no currently non-invasive specific serum biochemical marker of synovial tissue turnover [2].

The major organic components of the extracellular matrix of synovial tissue, are type I and III collagens, non-collagenous proteins and minor collagen molecules including type IV which is also a main protein of the blood vessel membrane, [6–9]. This biochemical composition makes challenging the development of synovial-specific biochemical marker assays based on the detection of circulating fragments of a single protein, because none of them is preferentially localized in this tissue. One way to improve tissue specificity is to measure the metabolism of two or more synovial matrix components. A complementary strategy is to detect neoepitopes of these proteins generated by catabolic enzymes upregulated in RA synovium [10].

The aim of this study was to develop a sensitive serum immunoassay assay of both type III and IV collagen degradation products (Col 3–4) which are released by MMPs, as a novel integrated marker of synovial tissue activity. Serum Col 3–4 was also measured in patients with RA with low or moderate disease activity and levels were compared to those of healthy controls.

## Methods

### RA patients

Sixty-six patients with RA participating in three clinical trials were investigated. Inclusion criteria included age 18 years and older, RA diagnosis according to the criteria of the ACR/ EULAR 2010, and a disease activity score 28 with erythrocyte sedimentation rate (DAS28-ESR) ≤ 3.2 for at least 6 months in the first study. In the two other studies, similar inclusion criteria were used, but patients were resistant to conventional treatments and required biotherapy (DAS28-ESR ≥ 3.2) according to EULAR recommendations. In all studies, patients with concomitant treatment with zoledronic acid or denosumab were excluded. All subjects gave informed written consent to participate in the study. This study was carried out in accordance with the Declaration of Helsinki. Oral and written informed consent was obtained from all participants. The study protocols were approved by the « Comité de Protection des Personnes Lyon, Sud-Est 1» N° 2015-A00655-44, Role of the ANS Dysregulation in the Persistence of Fatigue in Rheumatoid Arthritis Patients Treated with Anti-TNF (ANSRA) NCT02475486. Bone Microarchitecture Abatacept (BMA2) NCT02675218. Impact of the Persistence of Inflammation at Doppler Ultrasound Level on the Structural Evolution of Erosion in Rheumatoid Arthritis Treated with Biotherapy. NCT02531061.

### Healthy controls

Seventy healthy subjects matched for gender and age with patients with RA were used as controls. There were 53 women and 17 men with a mean ± SD age of 59 ± 11 years. Healthy men were randomly selected from subjects aged from 20 to 87 years who participated in a large prospective cohort study (STRucture of the Aging Men’s BOnes, STRAMBO) designed to explore the quantitative computed tomography bone microarchitecture parameters in men [11]. Healthy women were participants of a prospective study on the determinants of bone loss (Os des FEmmes de LYon, OFELY) including a total of 1039 subjects from 30 to 80 years of age These cohorts have previously been described in details elsewhere [12]. All subjects were healthy without any disease or treatment that could interfere with bone or joint metabolism, including hormone replacement therapy in postmenopausal women. The studies were approved by the local ethics committee. Written informed consent was obtained from all participants.

### Collagen type III and IV fragments assay (Col3-4 ELISA)

#### Production of polyclonal antibodies

Free synthetic ^178^-PPGPPGPhypGTS-^188^ (Col3-4) peptide derived from the sequence of the α1 chain of human type III collagen (SwissProt accession no. P02461), biotinylated and keyhole limpet hemocyanin (KLH)–coupled peptides were synthesized using standard 9-fluorenylmethoxycarbonyl solid-phase peptide synthesis [13] by ProteoGenix SAS (Schiltigheim, France). Two rabbits were immunised. On day 0, pre-immune sera were collected and each rabbit was injected intraperitoneally with 0.5 mg of KLH-conjugated Col3-4 peptide emulsified in equal volume with Freund’s complete adjuvant. Immunisations were repeated 6 times for 56 days (on days 14, 28, 35, 42, 49 and 56). Both rabbits were reinjected each time with 250μg of immunogen emulsified in Freund’s incomplete adjuvant as previously described [14]. At each bleeding (days 35 and 63), antisera were screened by microtiter to monitor the presence of antibodies recognising the Col3-4 peptide. On day 70, both rabbits were sacrificed to recover the antisera. Titration was performed by investigating the binding of subsequent dilutions of the antisera on microtiter plates coated with biotinylated Col3-4 peptide (see below). The titer was defined as the dilution of the antiserum giving 50% of the absorbance of the undiluted antiserum. Specific anti-Col3-4 antibodies were then purified against the Col3-4 peptide by standard immuno-affinity procedures. Both rabbits showed comparable immunological responses. Specific anti-Col3-4 antibodies from rabbit 1 were used for further development of the serum ELISA (see below).

#### Col3-4 ELISA protocol

Biotinylated Col3-4 peptide diluted in PBS-BSA buffer at pH 7.4 (200 μl of biotinylated Col3-4 peptide at 0.75 μg/L) was pipetted into each well of a Nunc Immobilizer Streptavidin plate (Thermo Fisher Scientific, Waltham, MA, USA) and was used as a standard. The plate was incubated for 2 hours at room temperature (RT) and then washed 5 times with a TBS buffer with 5 g/L of BSA and 0.05% (vol/vol) of Tween 20, pH 7.2. 50 μl of calibrator, or control, or serum samples, prediluted to one third with TBS buffer, was pipetted into each well and 50 μl of primary antibody (polyclonal antibody against Col3-4 peptide) diluted at 5.5 μg/L in TBS buffer was added into each well. After incubation for 18 hours at 4°C, the plate was washed 5 times and 100μl of a solution of peroxidase-conjugated goat anti-rabbit antibody (Jackson ImmunoResearch, West Grove, PA, USA) was pipetted into each well. The plate was incubated for 1 hour at RT, washed and 100 μl H_2_O_2_/tetramethylbenzidine substrate solution (Interchim, Montluçon, France) was pipetted into each well. After incubation at RT for 20 minutes in the dark, the colorimetric reaction was stopped by the addition of 100μl of 0.5M H_2_SO_4_, and the optical density at 450 nm corrected for the absorbance at 620 nm was measured. All samples were measured in duplicate.

### Specificity of Col3-4 ELISA for collagen sequences

The molecular specificity of the antibody used in the Col3-4 ELISA was investigated by competitive inhibition with the Col3-4 peptide used for immunization with the following Col3-4 derived peptides: 1) Col3-4 peptide extended by 1 amino acid at the C-terminal end (^178^PPGPPGPhypGTSG^189^); 2) Col3-4 peptide shortened by 1 amino acid (^178^PPGPPGPhypGT^187^) at the C-terminal end; 3) non hydroxylated Col3-4 peptide (PPGPPGPPGTS); 4) Human type III (α1) collagen unrelated peptide (^1144^PSGPPGKDGTS^1154^); 5) homologous Col3-4 peptide derived from the α5 chain of human type IV collagen (^1446^LQGPPGPPGTS^1456^); 6) homologous Col3-4 peptide derived from the α3 chain of human type IX collagen (^620^PQGPQGVPGTS^630^); 7) homologous Col3-4 peptide derived from the α1 chain of human type IV collagen (^392^EKGDRGFPGTS^402^). Native human type III collagen and recombinant type IV (α5) collagen were also tested.

### *In vitro* collagen digestion by MMP-9

*In vitro* tests of type III and IV collagen digestion by MMP-9 were performed based on the technique described by Sand and al. for type IV collagen digestion by MMP-12 [15]. Briefly, 200μg of native human type III collagen and 50μg of recombinant type IV (α5) collagen (Aviva Systems Biology Corporation, CA, US) were incubated in test-tubes for 48h at 37°C with 5μg of active human recombinant MMP-9 (Merck, Saint-Quentin-Fallavier, France) in MMP buffer. The reaction was stopped using ethylenediaminetetraacetic acid to a final concentration of 1 μM.

### Immunohistochemistry analyses

Human RA synovium biopsy was collected immediately after knee surgery in sterile conditions. The biopsy was fixed and processed according to standard procedures, embedded in paraffin and cut in sections of 5μm. Serial sections were deparaffinised, rehydrated and then treated for immunohistochemistry with specific primary polyclonal antibodies for human type III and type IV collagens (generous gift from NOVOTEC SAS, Lyon, France), Col3-4 collagen peptide and human MMP-9 (Abcam, Cambridge, UK) or rabbit IgG isotype as a negative control (Abcam, Cambridge, UK). Polink HRP plus rabbit AEC (GBI, Bothell, WA, USA) was used as a detection system. All antibodies tested including the anti-Col3-4 antibody were used at a concentration of 1μg/ml, except for the anti-MMP-9 antibody which was used at the 1/1000 dilution as recommended by the manufacturer. Briefly, deparaffinised sections were treated with endogen peroxidase blocking reagent (3% H_2_O_2_) for 10 min, the antigens were uncovered by incubation for 1 h at 80°C in citrate buffer (pH 6.0) and the non-specific sites were blocked with the protein block reagent (GBI Labs). Sections were then incubated overnight at 4°C with the different specific antibodies. This incubation was followed by a 30-min incubation with rabbit antibody enhancer and then with polymer horseradish peroxidase (HRP) during 30 min using 3-amino-9-ethylcarbazole as a chromogen. Finally, the sections were counterstained with Mayer’s haematoxylin (Merck) and digital pictures were obtained using a computer assisted microscope.

### Statistical analysis

All data are expressed as mean ± standard deviation (SD) or median (minimum-maximum). Comparisons between groups were performed using parametric Student’s t-test after log transformation of the data for serum Col3-4, serum CRP and DAS28-ESR. Correlations were estimated by the non-parametric Spearman rank correlation coefficient. All statistical analyses were performed using XLSTAT software.

## Results

### Analytic performances of serum Col3-4 ELISA

**Fig 1:**
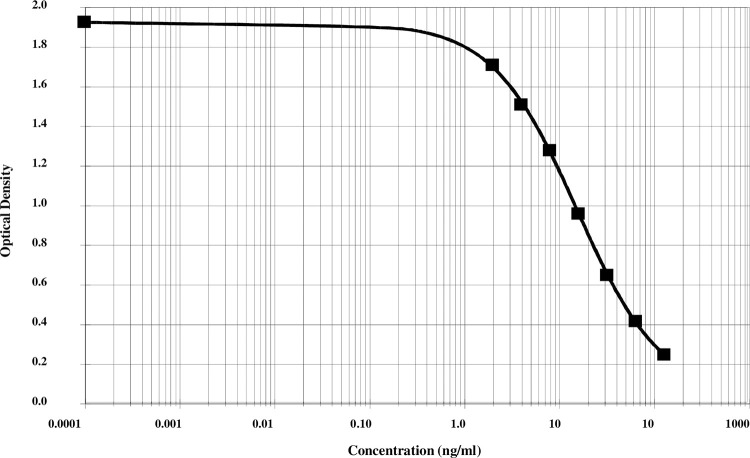
Example of Col3-4 ELISA calibration curve. A log-lin-4-parameter calibration curve is used to analyse the serum Col3-4 assay results. The Col3-4 concentration (ng/ml) of each sample is determined by interpolation from the standard curve. Four parameter logistic equation: y = b+ (a-b)/(1+xc)^d, a = 1,919, b = 0.059, c = 0.081, d = 0.952, R^2^ correlation coefficient = 0.999.

As shown in Fig 1, the calibration curve of the ELISA ranged from 0 to 128 ng/ml. The main analytical performances of the assay are listed in Table 1. Intra- and inter-assay coefficients of variation (CV) were assessed by 16 determinations of 4 different serum samples. Dilution recovery data were determined using 8 different serum samples diluted to 1/2 and 1/4 in sample buffer. Spiking recovery was determined by the addition of known quantities of standard peptides (20–40 ng/ml) into 3 different human serum samples. The stability of Col3-4 peptide levels was tested in 5 serum exposed for several hours at RT or 4°C, or subjected to freeze-thaw cycles.

**Table 1:** Summary of Col3-4 ELISA analytical performances.

**Assay precision**	Intra-assay CV < 10% (n = 16)
	Inter-assay CV < 15% (n = 16)
**Detection limits** ^**a**^	LLOD: 0.21 ng/ml
	LLOQ: 2.5 ng/ml
	ULOQ: 95 ng/ml
**Dilution recovery**	80–108%
**Spiking recovery**	88–125%
**Serum stability**	RT: > 4 hours
	4°C: > 6 hours
	Freeze-Thaw Cycles: > 4

^a^ LLOD, Lower Limit of Detection; LLOQ, Lower Limit of Quantification; ULOQ, Upper Limit of Quantification

### Specificity of the antibody used in the Col3-4 ELISA for collagen sequences

**Fig 2:**
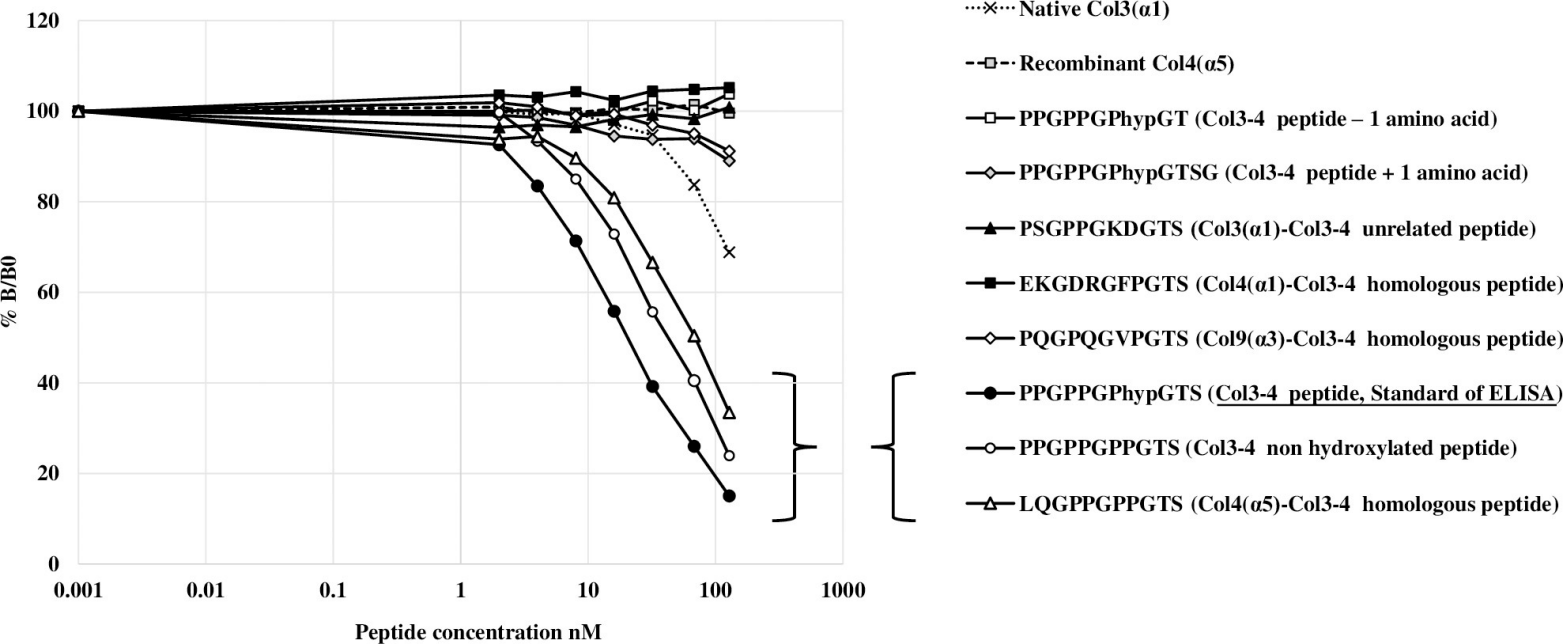
Specificity of the Col3-4 ELISA for collagen peptides and intact collagen molecules. The graph shows the competitive inhibitions between Col3-4 peptide, standard of ELISA with the following derived peptides for binding to the ELISA antibody 1) extended Col3-4 peptide (Col3-4 peptide + 1 amino acid), 2) shortened Col3-4 peptide (Col3-4 peptide—1 amino acid), 3) non-hydroxylated Col3-4 peptide (Col3-4 non-hydroxylated peptide), 4) unrelated type III peptide (Col3(α1)-Col3-4 unrelated peptide), 5) homologous type IV (α5) collagen peptide (Col4(α5)-Col3-4 homologous peptide), 6) homologous type IX (α3) collagen peptide (Col9(α3)-Col3-4 homologous peptide), 7) homologous type IV (α1) collagen peptide (Col4(α1)-Col3-4 homologous peptide). The graph shows also the recognition of the antibody with intact type III (α1) collagen (Native Col3 (α1)) and type IV (α5) collagen (Recombinant Col4 (α5). Y-axis shows the relative binding of the antibody at different concentrations of each molecule (B) expressed as a percentage of the binding with no competitor peptide (B0). The x-axis shows the molar concentration of each peptide. In brackets are the immunoreactive peptides.

The specificity of the antibody was studied by competitive inhibition experiments between the biotinylated Col3-4 peptide coated on the streptavidin plate and various free collagen molecules or peptides added in increasing concentrations (Fig 2). There was no significant cross-reactivity of the antibody with Col3-4 peptides that have been shortened or extended by 1 amino acid at the C-terminal end up to a concentration of 150 nM. The antibody recognized, but to a lesser extent, the sequence of Col3-4 peptide in which the hydroxyproline residue was replaced by a proline. The antibody also recognized with lower affinity a homologous sequence present in the α5 chain of human type IV collagen where 9 of the 11 amino acids at the C-terminal end were identical to the sequence of the Col3-4 peptide. The antibody did not show any significant immunoreactivity with an unrelated peptide sequence present in the α1 chain of human type III collagen sharing only the 3 amino acids at the C-terminal end with the original Col3-4 peptide sequence. The antibody did not cross-react with homologous Col3-4 peptides present in the α1 chain of human type IV collagen and in the α3 chain of human type IX collagen which share only the same quadruplet at the C-terminal end. No significant immunoreactivity was detected against native intact human collagen type III and recombinant human type IV α5 collagen chains. In summary, to be immunoreactive in the Col3-4 ELISA, the collagen fragments need to have a free serine residue at the C-terminal end indicating that the collagen molecules have to be cleaved by a proteinase to be detected. The hydroxylation of the proline residue in the sequence does not appear to be essential for recognition. More than 4 amino acids at the C-terminal identical to those of the Col3-4 sequence are also necessary for recognition, but do not provide full immunoreactivity.

### Immunohistochemistry analysis

**Fig 3:**
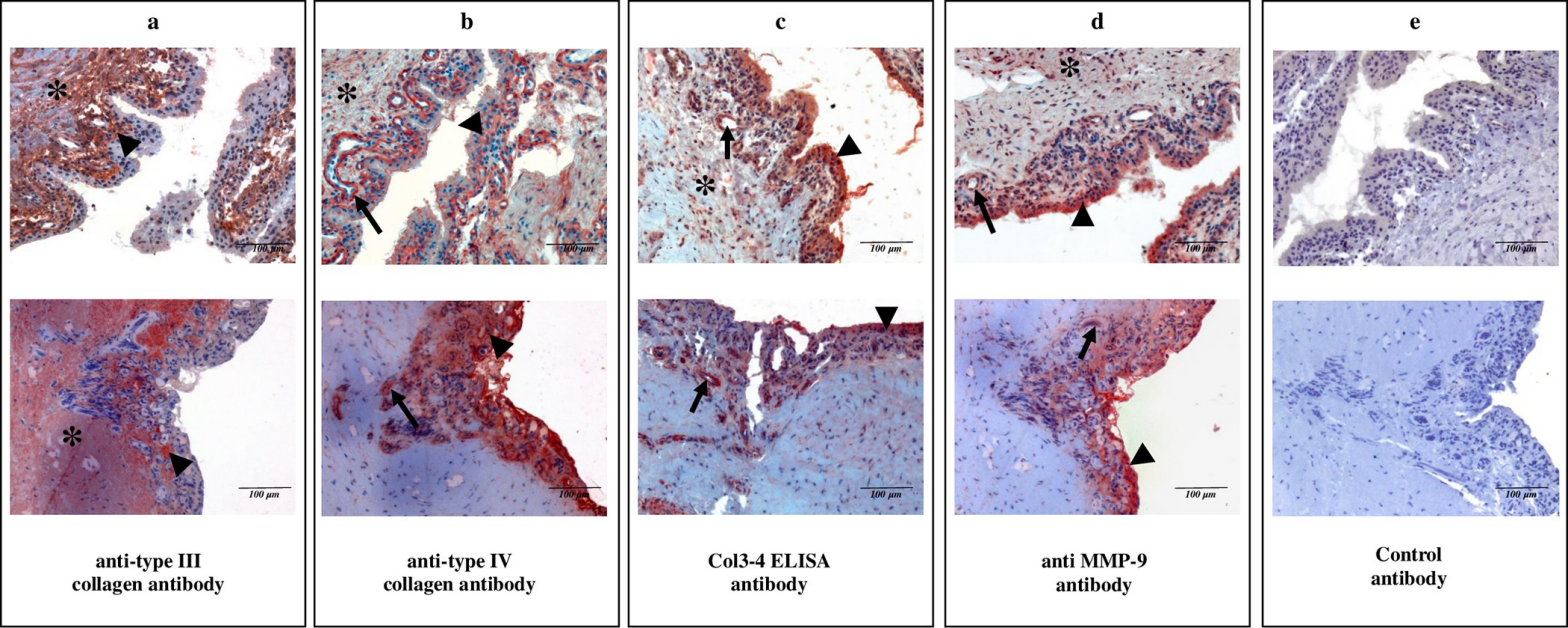
Immunohistochemistry of synovial tissue from a patient with RA. a) Staining of subintimal synovium (asterisk) and deeper area of cell lining layer (arrowheads) with anti-type III collagen antibody; b) staining of synovial intima (arrowheads), underlying vessels (arrows) and sub-intima (asterisk) with anti-type IV collagen antibody; c) staining of synovial intima (arrowheads), underlying vessels (arrows) and sub-intima (asterisk) with the polyclonal antibody used in the Col3-4 ELISA; d) staining of synovial intima (arrowhead), underlying vessels (arrows) and sub-intima (asterisk) with anti MMP-9 antibody; e) immunohistochemistry performed with rabbit IgG isotype control.

Fig 3 shows the immunostaining of a knee synovium section from a patient with RA by specific antibodies. The antibody of the Col3-4 ELISA showed a strong staining of blood vessels (arrows) which basement membrane is rich in type IV collagen. The capillaries lie just below or within the RA lining layer. The synovial lining layer where the destructive fibroblast-like and macrophage-like synoviocytes are located were also labelled by the antibody (arrowheads). The staining intensity of the sub-intima was faint (asterisk). A generic anti-collagen type III antibody mainly labelled the extracellular matrix in sub-intimal layers (asterisk). An anti-collagen type IV antibody strongly marked the capillaries (arrows), the lining layer (arrowheads) and to a lower extent the sub-intima matrix (asterisk). Staining with the anti-MMP-9 antibody was mainly located in the lining layer (arrowheads) and also around blood vessels (arrows), a distribution that was similar to that of the antibody used in the Col3-4 ELISA.

### COL3-4 is an MMP-9-mediated collagen neoepitope

**Fig 4:**
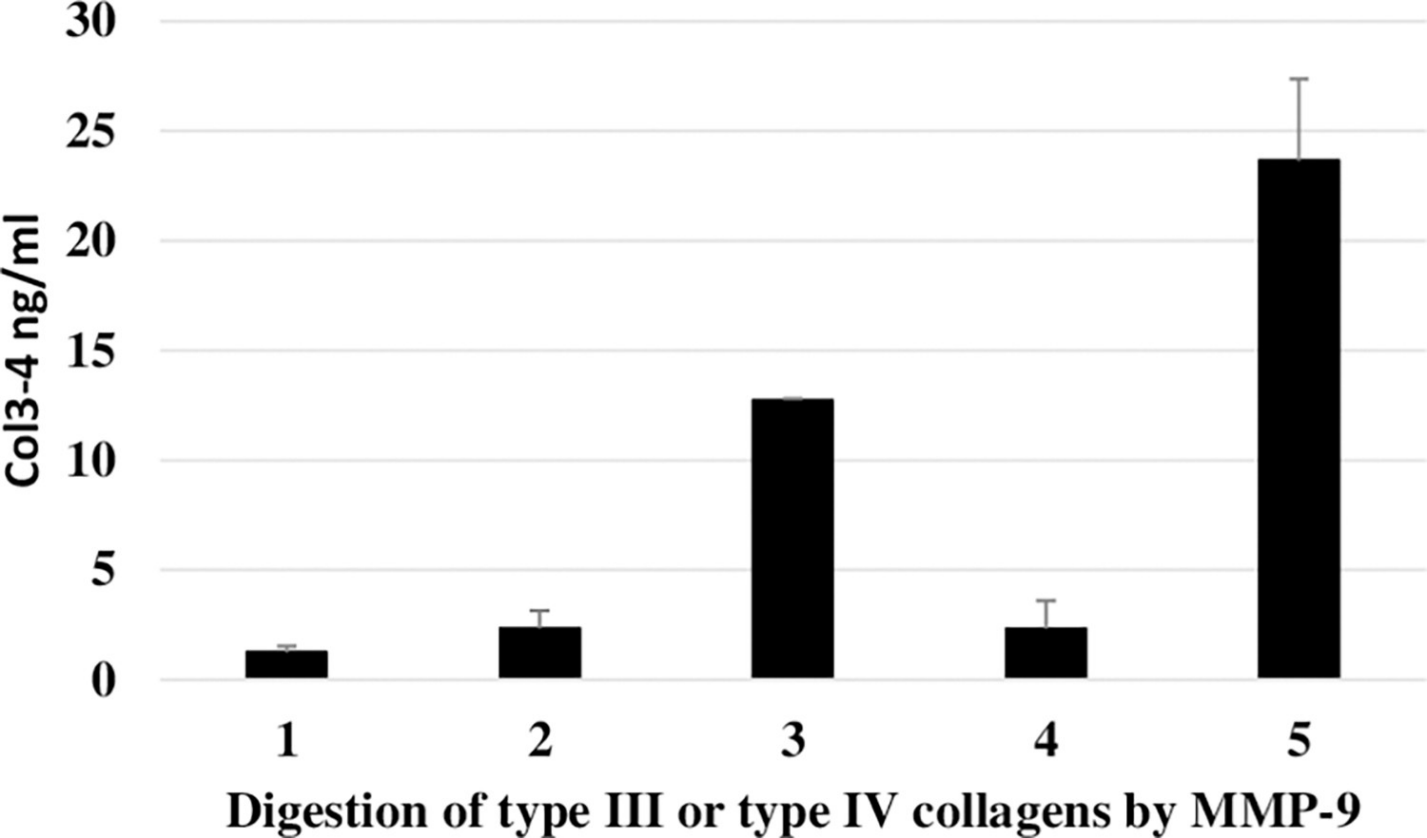
MMP-9 efficiently generated Col3-4 immunoreactivity from human type III and type IV collagen digestion. The graph shows the quantity of Col3-4 fragments measured by ELISA after incubation of human native type III collagen and recombinant type IV (α5) collagen with active recombinant human MMP-9 for 48h at 4°C. Results are shown as bars with mean ± SD of 2 independent experiments. 1) MMP buffer + MMP-9; 2) MMP buffer + native human type III collagen; 3) MMP buffer +MMP-9 + native human type III collagen; 4) MMP buffer + recombinant type IV(α5) collagen; 5) MMP buffer +MMP-9 + recombinant type IV(α5) collagen.

As shown on Fig 4, human recombinant MMP-9 efficiently generated Col3-4 fragments measured by ELISA from the digestion of both human native type III collagen and recombinant type IV (a5) collagen.

Immuno-histochemistry data are consistent with the *in vitro* findings. They suggest that the biological mechanisms responsible for the generation of the Col3-4 fragments in RA subjects are likely to involve MMP-9 produced by macrophage-type synoviocytes localized around blood vessels and the lining layer, regions containing both type III and IV collagens which act as a substrate for the enzyme.

### Serum Col3-4 levels in healthy controls and RA patients

**Fig 5:**
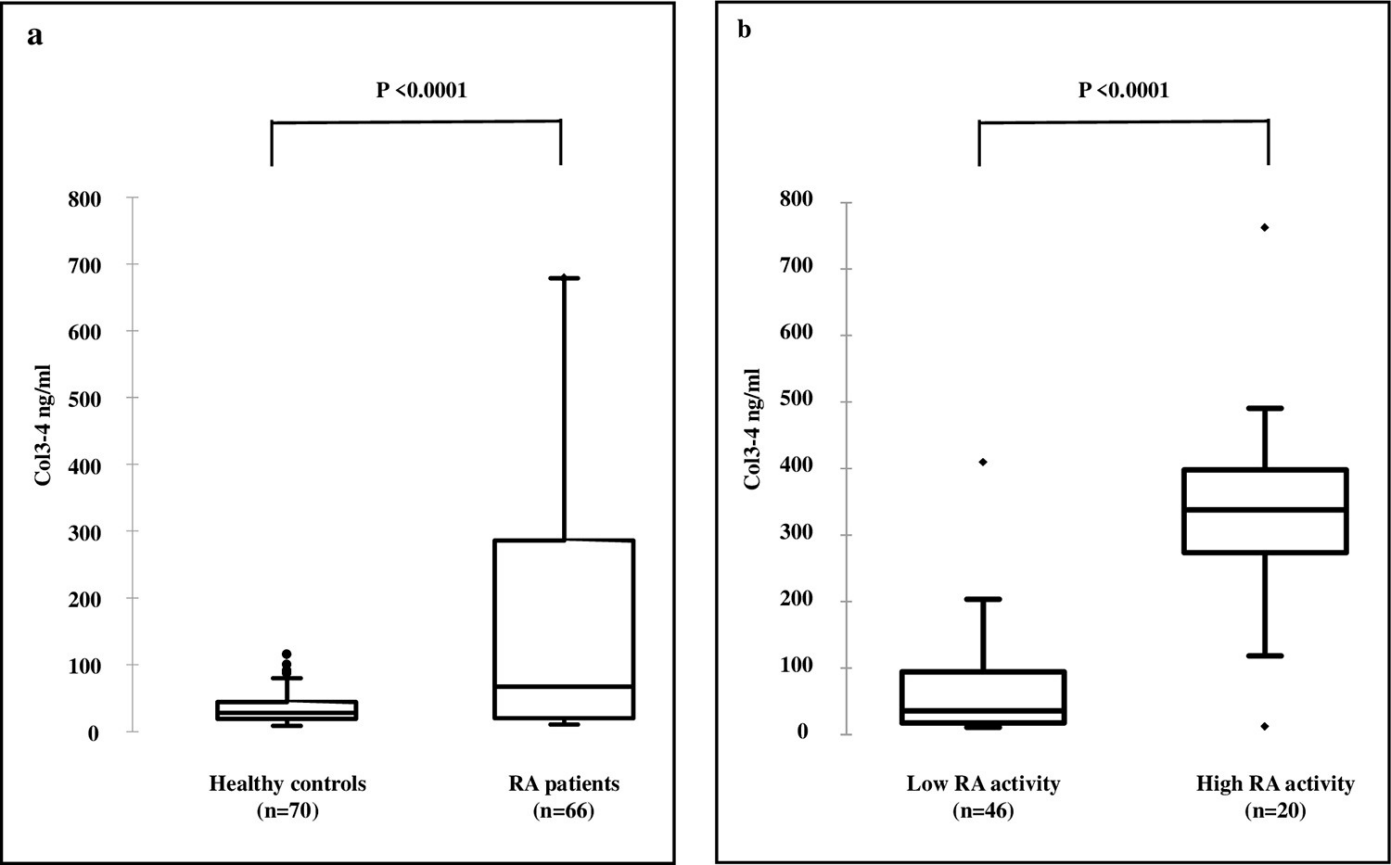
Box plots of serum Col3-4 levels in healthy controls and RA patients. a) Healthy controls vs RA patients; b) Patients with low-active *vs* high-active RA disease (DAS28-ESR cut-off ≥ 3.2). The upper and lower limits of the box represent the 75 and 25 percentiles of the distribution respectively. The horizontal bar in the box is the median values of each group.

Table 2 shows the characteristics of healthy controls (n = 70) and patients with RA (n = 66). Patients with RA were divided in two groups according to the disease activity assessed by the DAS28-ESR score with a cut-off value of ≥ 3.2. The gender ratio was similar in the three groups of subjects with a larger proportion of women, as expected. Patients with RA and higher disease activity were older than those with lower DAS28-ESR and healthy controls. Body mass index was slightly higher in patients with RA than controls, but similar in the two groups of RA subjects. There was no significant correlation between Col3-4 levels and age or BMI, both in healthy subjects and patients with RA (*p* > 0.05 for all correlations). Median Col3-4 levels were significantly higher in patients with RA compared to healthy controls (+ 240%; *p* < 0.0001) (Fig 5). Levels of serum Col3-4 were also significantly higher in the group of RA patients with high than in those with low disease activity (+ 896%; *p* < 0.0001), with virtually no overlap in the distribution of the values between the two groups (Fig 5). When all patients with RA were analysed together, there was a significant correlation between serum Col3-4 levels and CRP (*r* = 0.76; *p* < 0.0001) or DAS28-ESR (*r* = 0.53; *p* < 0.0001, S1 Fig).

**Table 2:** Demographic variables, DAS28-ESR, serum CRP and Col3-4 in healthy controls and in patients with RA with low or high disease activity.

	Gender (F/M)	Age (year) ^a^	BMI (kg/m^2^) ^a^	DAS28- ESR score ^b^	CRP (mg/L) ^b^	Col3-4 (ng/ml) ^b^
**Healthy controls (n = 70)**	53/17	59.1 ± 11.1	23.9 ± 4.3			28 [8.7–100]
**RA patients (n = 66)**	48/18	62.0 ± 10.9	26.4 ± 5.4	2.6 [0.5–6.1]	4.0 [0.3–103]	68 [11–679]
**RA patients DAS28-ESR <3.2 (n = 46)**	36/10	59.5 ± 9.3	26.9 ± 5.9	2.3 [0.5–3.1]	2.2 [0.3–55]	36 [11–410]
**RA patients DAS28-ESR ≥3.2 (n = 20)**	12/8	67.6 ± 11.3	25.4 ± 4.2	4.9 [3.2–6.1]	29 [0.3–103]	320 [12–679]

^**a**^ Mean ± SD

^**b**^ Median [Minimum-Maximum]

When results were analysed by gender, men and women in the healthy group were age-matched to those in the RA group. In women, BMI of controls was lower than that of RA patients. The Col3-4 values were significantly increased in women and men with RA compared to corresponding gender-matched healthy controls. In both healthy controls and RA patients, Col3-4 values were higher in men than in women (Table 3). In the RA group, women were slightly younger than men but BMI were comparable regardless of gender. Col3-4 and CRP levels were increased in men and women with active disease (Table 4).

**Table 3:** Demographical data and serum Col 3–4 levels in healthy controls and in patients with RA by gender.

	Gender	N	Age (year)^a^	BMI (kg/m2) ^a^	Col3-4 (ng/ml) ^b^
**Healthy controls**	F	53	56.9 ± 10.7	23.0 ± 3.3	25 [8.7–100]
	M	17	65.9 ± 9.6	27.1 ± 5.3	40 [11–87]
**RA patients**	F	48	59.6 ± 9.5	26.5 ± 6.2 ^†^	47 [11–679] ^†^
	M	18	68.4 ± 12.2	26.8 ± 3.8	144 [14–388] ^†^

^**a**^ Mean ± SD

^**b**^ Median [Minimum-Maximum]

^†^ *p* < 0.001, RA patients *vs* gender-matched healthy controls

**Table 4:** Demographical and biological variables in patients with RA with low and high disease activity by gender.

	Gender	N	Age (year) ^a^	BMI (kg/m2) ^a^	DAS 28-ESR score ^b^	CRP (mg/L) ^b^	Col3-4 (ng/ml) ^b^
**RA patients DAS28-ESR <3.2**	F	36	58.0 ± 9.2	25.9 ± 6.4	2.3 [0.5–3.1]	2.1 [0.3–55]	29 [11–410]
	M	10	65.0 ± 11.2	26.9 ± 3.4	2.1 [0.6–3.0]	3.1 [0.8–22]	63 [18–283]
**RA patients DAS28-ESR ≥3.2**	F	12	64.3 ± 9.3	27.8 ± 5.1	4.9 [3.5–6.1]	26 [0.7–103]	350 [12–679]
	M	8	72.6 ± 12.7	24.8 ± 3.8	4.7 [3.2–6.0]	41 [0.3–68]	288 [14–388]

^**a**^ Mean ± SD

^**b**^ Median [Minimum-Maximum]

## Discussion

In this report, we describe the development of a sensitive ELISA measuring circulating fragments of type III and type IV collagen which originated during the degradation of connective tissues including the synovium that was named Col3-4. Serum levels of this novel biochemical marker were significantly increased in patients with RA compared to healthy controls, values increasing with higher degree of disease activity. This suggests that serum Col3-4 may be a useful non-invasive index of active synovium.

The Col3-4 ELISA demonstrated adequate analytical performances in terms of sensitivity, accuracy, repeatability, dilution recovery, spiking recovery and analyte stability. This indicates that this ELISA is suitable to obtain reliable and reproducible results in human clinical studies.

An important feature of immunological assays is the specificity of the antibody used to detect the biochemical marker. The antibody was produced using as immunogen a 11 amino-acid peptide which is specific of human type III collagen. The inhibition experiments demonstrated that to be immunoreactive in the ELISA, the peptides require the presence of a free serine (S) residue at the C-terminal end. This indicates that fragments detected by the ELISA are neo-epitopes generated from proteolytic cleavage of the collagen molecule. In agreement with this hypothesis, we found no cross-reactivity of Col3-4 antibody with intact native human type III collagen and recombinant full-length type IV collagen. However, the sequence of the Col3-4 peptide in which the hydroxyproline was replaced by a proline and the homologous sequence of type IV (α5) collagen which have several amino acids in common with the Col3-4 peptide at the C-terminal end were also immunoreactive in the ELISA but with lower affinity. In contrast another type III collagen peptide that shares only the final GTS triplet in common with the Col3-4 sequence was not detected. The presence of a GTS motif at the C-terminal end is thus not sufficient for antibody recognition but the proline (or preferably its hydroxylated form) in position just before this triplet is critical to the immunoreactivity. Still this 4-amino-acid motif does not constitute the full epitope of the antibody as type IV (α1) and type IX (α3) collagen peptides that share the final PGTS quadruplet in common with Col3-4 sequence were not detected. Interestingly, sequence analysis of other main collagens of connective tissues including type I (bone) and type II (cartilage) collagens, shows that the critical PGTS motif is not present indicating that putative fragments of these two molecules will not be detected by the ELISA.

The Col3-4 ELISA does detect fragments originating from type IV collagen in addition to the target type III collagen molecule. Type IV collagen is a major constituent of the basement membrane that exists in three distinct α-chain trimers: α1α2α1, α3α4α5, and α5α5α6. The α1α2α1 trimer, is present in the basement membrane of all tissues while the others isoforms are minor and have more restricted tissue distribution [16]. Because the widely distributed α1α2α1trimer was not immunoreactive in the ELISA, Col3-4 may be more tissue specific.

In normal and RA synovial tissue, type IV collagen including the α5 chain containing isoforms, are present in the matrix that surrounds the synoviocytes of the lining layer as well as in the basement membrane of blood vessels [6, 17–19]. MMP-9 has known proteolytic activity on both type III and type IV collagen containing tissues [20]. In the RA synovium, MMP-9 is highly expressed in infiltrating leukocyte cells, vascular endothelial cells, and intimal lining synoviocytes [19, 21, 22]. In the synovial fluid of patients with RA, MMP-9 levels have shown to be increased and were associated with vascular endothelial growth factor supporting a role of this enzyme in the formation of the highly neovascularized synovial pannus [21, 23]. Using indirect immunofluorescence double-labeling techniques, Poduval et al. [19] demonstrated co-labeling of type IV collagen and MMP-9 in neovascularized, fibroblast- and macrophage-rich synovial lining of RA. They observed that type IV collagen immunostaining is decreased in RA compared with traumatic synovium, whereas MMP-9 expression is strongly increased, suggesting an intense proteolytic activity especially in the intimal regions of the RA synovium [19]. Increased levels of MMP-9 measured by ELISA have also been reported in the serum and plasma of RA patients compared to healthy controls [21, 24]. These data indicate that MMP-9 plays an important role in the physiopathology of RA and the remodeling of the synovial collagen matrix. Our immunohistochemistry experiments of RA synovial tissue showed that the Col3-4 antibody stained the extracellular matrix surrounding the synovial cells and blood vessels and confirmed the presence of MMP-9 in these localizations. In test-tubes studies, we also found that the digestion of human type III and type IV collagen by active recombinant MMP-9 efficiently generated Col3-4 immunoreactive fragments measured by ELISA. All together, these data suggest that Col3-4 immunoreactivity in the synovial tissue of patients with RA results from the degradation of type III and type IV collagens by MMP-9 -and possibly other proteinases- which is over-expressed in destructive fibroblasts of the synovial lining layer [25].

The ELISA for serum Col3-4 may be an integrated indicator of the catabolism of two of the collagens which are both implicated in RA-related synovial tissue alterations. We cannot however exclude that the Col3-4 assay also detects circulating collagen fragments derived from the remodelling of type III/IV collagen containing non-synovial tissues. Type IV collagen localizes in the basal membrane of several tissues and particularly in blood vessels. Type III collagen is also present in the extracellular matrices of various organs. This lack of tissue specificity is a limitation of almost all biochemical markers reflecting the remodeling of extracellular matrices including the validated type I collagen markers approved by the health authorities for osteoporosis management [26].

The Col3-4 assay has however several features which may improve its specificity for the active RA synovial tissue compared to existing markers. It is based on the concomitant immunodetection of fragments from two collagens which are present in the synovium membrane, including a minor isoform of type IV that has a more restricted tissue distribution than the major form. It is a collagen neoepitope generated by MMP-9 which is highly expressed in the synovial tissue of active RA patients. Thus Col3-4 can be released only from tissues containing both type III collagen and the α5 type IV collagen isoforms and that have the proteolytic machinery able to generate this specific neoepitope. Col 3–4 is also likely to represent an integrated marker of the various biological mechanisms which lead ultimately to the dysregulated synovial tissue turnover. In contrast, the measurement of selective MMPs in the serum reflects the contribution of almost all collagen-containing tissues and is an index of only one of the various proteolytic pathways involved in matrix remodeling. From a technical standpoint, MMPs circulate in various molecular entities (pro-MMP, active MMP, MMP bound to inhibitors) and their relative concentration may vary according to the clinical conditions. It is unclear which of these different forms is (are) the most relevant in RA. Because these technical limitations in serum MMPs assays, we believed it was more informative to investigate the direct association of Col3-4 with MMP-9 at the synovial tissue level. In addition, the available volume for was not sufficient to perform MMP-9 measurement in this study.

Other putative biochemical collagen markers of synovial tissue have been described. Increased urinary levels of glucosyl galactosyl pyridinoline, which cross-links type I and III collagens, has been shown to be associated with an increased risk of progression of joint destruction in early RA [27]. ELISAs have been developed to measure other fragments of type III (C3M) or type IV (C4M) collagens, but not the two collagens in the same assay. They were primarily developed as biochemical markers of liver fibrosis [15, 20]. In patients with long standing RA participating in randomized clinical trials of tocilizumab, serum C3M and C4M have been reported to be associated with disease activity and be useful to monitor clinical efficacy of the treatment, but did not predict radiographic progression. C3M was also not related to the erosion phenotype in a small retrospective study of patients with early RA [28].

Ultimately the clinical value of biochemical markers resides in the association of their serum concentrations with relevant clinical outcomes. In a preliminary clinical study including limited but well characterized patients with RA participating in clinical studies, we found that serum Col3-4 levels were significantly increased in patients with RA compared to gender and age matched healthy controls participating in epidemiological studies and recruited in the same geographical area as the RA subjects. Serum Col3-4 levels were significantly associated with the disease activity score DAS28-ESR. Individuals with a DAS28-ESR ≥3.2 values had markedly elevated Col3-4 levels compared to both healthy controls and RA subjects with low disease activity with virtually no overlap in the distributions. Factors other than disease activity may influence the concentration of serum Col3-4 such as renal and liver functions and concomitant therapies which also affect the measurement of several other serum markers. In this study, none of the healthy controls and subjects with RA had severe renal insufficiency or liver diseases. Only 12 patients with RA were receiving glucocorticoid and we could not detect a statistical difference in Col3-4 levels compared to non-users although we cannot exclude a minor influence because of the small number of users. The primary objective of this study was to develop an immunoassay for a novel biochemical marker based on qualified biological reagents, to validate its technical performances, to characterize its synovial tissue localization and to provide preliminary clinical data in patient with RA. The pre-analytical factors that may affect the circulating levels of Col3-4 will be investigated in further investigations.

In subjects with low RA disease activity, although on average Col3-4 levels were increased, a significant proportion of subjects had values within the normal range. This indicates that this biochemical marker, like the other tissue remodelling markers investigated so far, has a modest diagnostic value in treated subjects with low disease activity. More likely the measurements of Col 3–4 in patients with early RA could be useful to identify individuals with high synovium activity who are more likely to progress (prognostic value) and/or respond to specific treatments (monitoring efficacy). Larger longitudinal studies using radiography or MRI to document joint damage progression are needed to validate this hypothesis.

## Supporting information

S1 Fig Scatter plot of Col3-4 *vs* DAS28-ESR in RA patients. (TIF)
