# Supplementary figures and images for: Serum Col3-4: A new type III and IV collagen biochemical marker of synovial tissue turnover in patients with rheumatoid arthritis
Source: PLoS One. 2023 Apr 13;18(4):e0282954. doi: 10.1371/journal.pone.0282954 (PMC10101524; doi:10.1371/journal.pone.0282954)

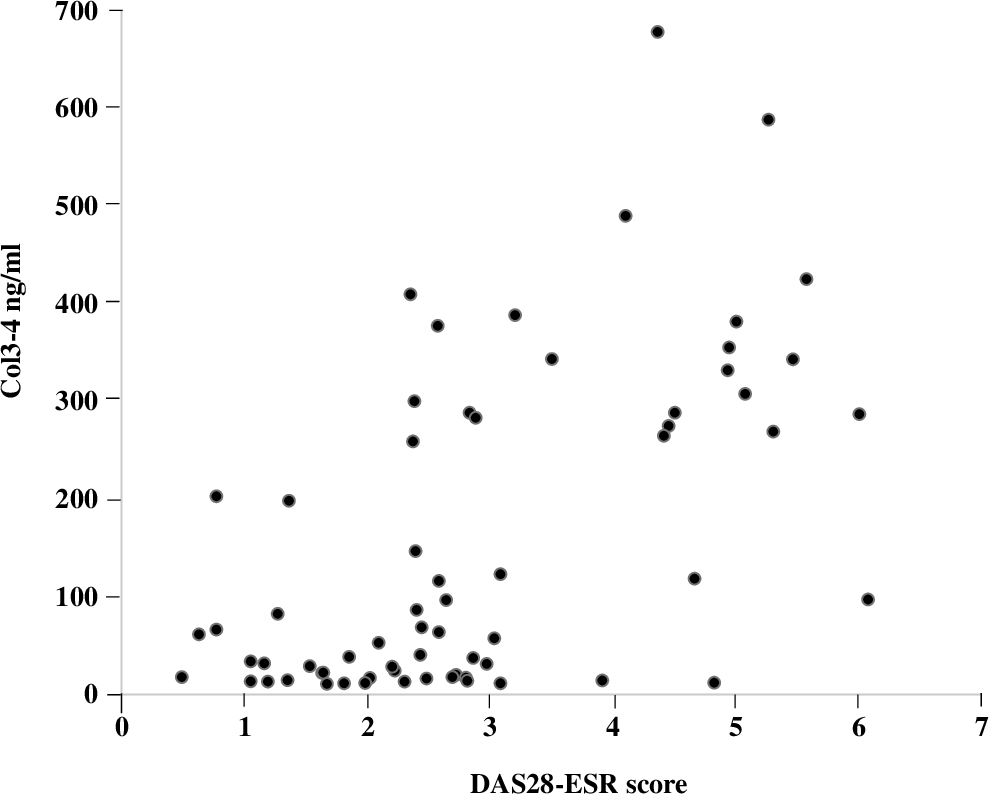

Supplement: S1 Fig — (TIF) [file pone.0282954.s001.tif]
